# Cyclophosphamide-induced lung damage in mice: protection by a small preliminary dose.

**DOI:** 10.1038/bjc.1980.167

**Published:** 1980-06

**Authors:** C. H. Collis, C. M. Wilson, J. M. Jones

## Abstract

Cycylphosphamide (Cy) produces an interstitial pneumonitis in CBA mice. The extent of the lung damage has been quantified by measuring the increase in ventilation rate over 6 weeks after an i.p. injection of Cy 200, 250 and 300 mg/kg. A dose-dependent response was found. When a preliminary ("priming") dose of Cy at 50 mg/kg was given 7, 9 or 14 days before a single large dose of 250 mg/kg, lung damage was reduced, as shown by a smaller increase in ventilation rate than in those receiving 250 mg/kg alone, and this difference was significant (P less than 0.01) in the Day-14-and highly significant (P<0.001) in the Day-7-"primed" groups. When primed less than 7 days before, there was a relative increase in ventilation rate, which was statistically significant (P less than 0.01) in the Day-1-primed group. Similar effects were also seen in the survival of the mice.


*Br. J. Cancer* (1980) **41**, 901

### CYCLOPHOSPHAMIDE-INDUCED LUNG DAMAGE IN MICE: PROTECTION BY A SMALL PRELIMINARY DOSE

C. H. COLLIS, C. M. WILSON AND J. M. JONES*

*From the Radiotherapy Research Unit, Divisions of Radiotherapy and Biophysics, Institute of Cancer Research, Sutton, Surrey SM2 5PX, and the *Division of Epidemiology, Institute of Cancer Research, Sutton, Surrey SM2 5PX*

Received 15 January 1980 Accepted 20 February 1980

**Summary.—Cyclophosphamide (Cy) produces an interstitial pneumonitis in CBA mice. The extent of the lung damage has been quantified by measuring the increase in ventilation rate over 6 weeks after an i.p. injection of Cy 200, 250 and 300 mg/kg. A dose-dependent response was found. When a preliminary ("priming") dose of Cy at 50 mg/kg was given 7, 9 or 14 days before a single large dose of 250 mg/kg, lung damage was reduced, as shown by a smaller increase in ventilation rate than in those receiving 250 mg/kg alone, and this difference was significant ($P < 0{\cdot}01$) in the Day-14- and highly significant ($P < 0{\cdot}001$) in the Day-7-"primed" groups. When primed less than 7 days before, there was a relative increase in ventilation rate, which was statistically significant ($P < 0{\cdot}01$) in the Day-1-primed group. Similar effects were also seen in the survival of the mice.**

INTERSTITIAL PNEUMONITIS and pulmonary fibrosis resulting from cyclophosphamide (Cy) administration in man is well documented, although it is rare (André *et al.*, 1967; Patel *et al.*, 1976; Mark *et al.*, 1978). The use of Cy may however pose more of a clinical problem by enhancing radiation lung damage (Phillips & Fu, 1976). Cy has also been reported to produce consistent lung damage in rats after a single i.p. dose of 200 mg/kg (Gould & Miller, 1975). The histological changes seen in the rat were similar to those seen in man, although the damage occurred within a few days, unlike that in man, which usually occurs after several months of administration.

By giving a preliminary small dose of a cytotoxic agent at a specific interval before a subsequent large dose of the same or a different cytotoxic agent, the damage to normal tissues may be reduced. This procedure will be called "priming", and the preliminary small dose referred to as the "priming dose". Priming with Cy has been shown to reduce lethality in mice after total-body irradiation, by enhancing haemopoietic recovery (Gregory *et al.*, 1971; Millar & Hudspith, 1976; Blackett & Aguado, 1979). Cy priming acts by a similar mechanism to protect against busulphan toxicity (Millar *et al.*, 1975). Cy priming has also been shown to increase mouse survival after a second large dose of Cy, but neither the cause of death nor the mechanism of protection could be explained (Millar & McElwain, 1978).

This is the first study in which the ventilation rate has been used as a functional assessment of the extent of drug-induced lung damage. So far as we are aware, there have also been no previous reports of protection of the lung from the toxic effects of anticancer agents by priming with a cytotoxic drug. Although pulmonary oedema has been reported following Cy in BALB/c mice (Berenbaum, 1974) Cy-induced interstitial pneumonitis has not been previously described in the mouse.

### METHODS AND MATERIALS

Twelve-week-old CBA male mice were used throughout the study. Groups of 5 mice were given Cy monohydrate (Koch-Light Laboratories) at 200 mg, 250 mg and 300 mg/kg by i.p. injection. Further groups of 5 mice were given a priming dose of 50 mg/kg Cy, from 14 days to 12 h before a second dose of 250 mg/kg. A preliminary experiment had been carried out, in which the second large dose was 300 mg/kg.

The ventilation rate was measured several times a week using a whole-body mouse plethysmograph as described by Travis *et al.* (1979*a*). The mouse was placed in a perspex chamber of 250 ml with a microphone sealed into it at one end, and a sealed door at the other end. Air was drawn through the chamber at 100 ml/min. The signal from the microphone was amplified and then passed through filters to exclude signals outside 1–10 Hz, the range of ventilation rates likely to be encountered. The output was passed through a zero-crossing rate meter and displayed by a pen recorder.

The data obtained over the first 5 weeks were analysed by a two-way analysis of variance. For each treatment the ventilation rates were examined over weekly periods. The differences between these periods and between the treatment groups were analysed. No significant differences among the times of assessment were found, but significant differences among the treatment groups were. Each set of data was therefore averaged over the 5-week period, and *P* values obtained for differences between treatments, using Student's *t* test (see results).

Pairs of mice were killed before, and 1, 2, 3, and 4 weeks after a dose of 300 mg/kg of Cy. The lungs were inflated and fixed in formol-saline, and sections were stained with haematoxylin and eosin, Gordon and Sweet's stain for reticulin and van Giesen's stain for collagen. The red-blood-cell count was measured weekly.

### RESULTS

Ventilation rates were measured at regular intervals for 6 weeks after Cy administration. The results for 3 different dose levels are shown in Fig. 1. Control rates remained within the range 4–5 Hz. In all treatment groups there was a rise in ventilation rate within 3 days. The rate of rise during the first 7 days after treatment appeared to increase with dose, and beyond 2 weeks the ventilation rate in mice given the lowest dose (200 mg/kg) declined, while that for the highest dose (300 mg/kg) continued to increase. A significant difference ($P < 0{\cdot}001$) was found between the 200mg/kg- and 250mg/kg-treated groups.

FIG. 1.—Mean ventilation rates of groups of mice treated with cyclophosphamide at 200 mg/kg (□), 250 mg/kg (●) and 300 mg/kg (○) together with untreated controls (▲), measured over 6 weeks after injection. The lines through the means were drawn by eye.

The mean ventilation rates following 250 mg/kg preceded by a single 50mg/kg priming dose, given 12 h to 14 days beforehand, are shown in Fig. 2. In each panel of this figure the survival of the treated group of 5 mice is indicated: the results are clearly less reliable when few mice survived. Most of the curves show a first peak in breathing rate at 5–8 days after the large dose, and in some cases there is evidence of a second peak at around one month. The mean ventilation rates of those mice primed at 7, 9 and 14 days appear generally lower than those of the unprimed control group, shown in Fig. 2 as a dashed line. The mean rate of the 1-day-primed group appears higher.

The mean ventilation rates, averaged over the 5-week period, are shown in Fig. 3. It can be seen that there was a tendency for the mean ventilation rate to

FIG. 2.—Mean ventilation rates (±s.e.) of groups treated with a priming dose of 50 mg/kg, from 14 days to 12 h before a second large dose of 250 mg/kg cyclophosphamide. The dashed line in each graph represents the unprimed group. At the bottom of each graph are plotted the number of mice surviving. The control group is shown with the Day 14 primed group.

exceed the unprimed control value at short intervals, and to fall below it at longer intervals. The values at 7 and 14 days show significant protection ($P < 0{\cdot}01$) and the value at 1 day shows significant

FIG. 3.—Mean ventilation rate over 5 weeks (±s.e.) following 250 mg/kg cyclophosphamide primed by 50 mg/kg at the intervals shown.

enhancement of response ($P < 0{\cdot}01$). The change-over from enhancement to protection occurred at an interval of about 5 days. Similar trends have also been seen in another experiment, in which the large dose of Cy was 300 mg/kg.

The numbers of mice surviving at 6 weeks are shown in Fig. 4. Similar trends are seen in both experiments, with reduced lethality in those mice primed 7–14 days beforehand, and in those primed at 12 h. Also shown in Fig. 4 (lower panel) as a dashed line, are the mean ventilation rates over the first 5 weeks. The increased survival in the 7-, 9- and 14-day primed groups is mirrored by the reduced ventilation rates in these groups, and the poor survival in the group primed at 1 day agrees well with the increased ventilation rate of this group. It can also be seen that

FIG. 4.—Numbers of mice surviving at 6 weeks after a large dose of cyclophosphamide (300 mg/kg in the top panel, and 250 mg/kg in the bottom panel) both primed with 50 mg/kg cyclophosphamide at the time intervals shown. The dashed line represents the mean ventilation rates over 5 weeks (from Fig. 3).

the increased survival of the 12h-primed group is associated with a reduction in ventilation rate, compared to the Day-1-primed and unprimed groups.

Histological sections of the lung were examined by light microscopy at 1, 2, 3 and 4 weeks after Cy 300mg/kg. At 1 week there were the typical features of an acute interstitial pneumonitis. There was a cellular infiltrate which primarily consisted of neutrophil and basophil leucocytes and plasma cells. There was some hyperplasia of the granular pneumocytes. Many of the cells appeared atypical and the nuclei were hyperchromatic. The interalveolar walls were thickened, not only by increased cellularity, but also by interstitial oedema. In some areas there was alveolar collapse, although for the most part the alveolae were patent and intact.

At 2 weeks, although there was a slight decrease in the overall cellularity due to a reduction in the cellular infiltrate, there was further hyperplasia of the granular pneumocytes, many appearing enlarged and with foamy cytoplasm, and some of these cells sloughing into the alveolar spaces. Segmental areas of collapse and consolidation in the right lower lobe were noted in all the mice killed at 2, 3 and 4 weeks. The alveolar spaces in these areas were filled with cellular debris and sloughed epithelial cells. However, there was no evidence of any polymorphonuclear leucocyte infiltrate. Organization and early fibrosis were mostly seen in these areas, and were most marked at 3 and 4 weeks after the drug, although the overall cellularity was slightly reduced at this later time.

The red blood cell count was measured at 1, 2, 3 and 4 weeks after cyclophosphamide 300 mg/kg, and no evidence of anaemia was found.

### DISCUSSION

The findings of marked histological changes similar to those reported in rats (Gould & Miller, 1975) confirms that Cy induces pneumonitis in mice as well as in rats and man (Patel *et al.*, 1976). Although fibrosis was not prominent in these mice, they were only examined up to 4 weeks after administration, and fibrosis may develop later. It was also found that individual mice nearly always have an increase in ventilation rate before death, which would suggest, if the increase in ventilation rate is due to lung damage, that lung damage is the cause of death.

The evidence that an increase in ventilation rate is related to Cy-induced lung damage is as follows. Firstly, the onset of tachypnoea coincides with the development of histological pneumonitis. Secondly, no other causes of tachypnoea, in particular no evidence of anaemia, were found. However, cardiac damage, which has been reported in dogs (O'Connell & Berenbaum, 1974) was not specifically excluded. In the dogs, however, the drug was used in a considerably more toxic range, since the dogs died within a few hours of administration, rather than days, as in these mice. Thirdly, other agents causing lung damage, such as bleomycin (unpublished data) and irradiation (Travis *et al.*, 1979*a*) also increase in ventilation rates, but drug toxicity or sickness without lung damage causes a slowing of ventilation rather than tachypnoea.

Further evidence indicates a quantitative as well as a qualitative relationship of ventilation rate to lung damage, *i.e.* that the extent of lung damage is related to the ventilation rate. A scored assessment of histological damage after lung irradiation has shown that the ventilation rate correlates well with the extent of histological damage (Travis *et al.*, 1979*b*). In the present study, the highest ventilation rates are seen shortly before death, when lung damage is maximal. Furthermore, the mean ventilation rate closely follows the number of mice surviving (Fig. 4) and, if death is due to lung damage, the number of mice surviving reflects the extent of lung damage.

The results show that the mean ventilation rate, and therefore the extent of the lung damage, was related to the dose of drug administered, a highly significant difference in ventilation rates being found between the 200mg/kg and 250mg/kg doses. Although there is considerable overlap between the effects of 250 mg/kg and 300 mg/kg (as shown in Fig. 1), clearer separation was seen in earlier experiments.

An allergic response has been suggested as a mechanism for cytotoxic drug-induced lung damage in man, largely because of its sporadic occurrence and lack of evidence of dose-dependency in many instances. However, the histological changes seen with Cy, and indeed with most cytotoxic drug-induced lung damage in man, favours a direct toxic effect (Sostman *et al.*, 1977). The finding here of a dose-dependent response lends further support to a direct toxic effect, rather than an allergic response.

It was of interest to have examined a tissue, such as the lung, with a slow cell turnover, since most of the studies of normal-tissue protection have been carried out in rapidly proliferating tissues, such as marrow and intestine. Mortality due to bone marrow failure after treatment with melphalan and busulphan may be reduced by priming at 2 days with melphalan or cyclophosphamide respectively (Jeney *et al.*, 1968). This protection is due to enhanced recovery of the bone marrow (Millar *et al.*, 1975). After irradiation, similar protection of the marrow, assessed by the spleen CFU-S, is optimal with a slightly longer priming interval of 3 days with Cy, and 1 day after priming with cytosine arabinoside (Blackett, personal communication). Thus the optimal priming interval varies with the nature of the priming agent, and probably also with the principal toxic agent. It may also vary in different tissues. The gut is protected against irradiation by cytosine arabinoside, which acts by a different mechanism and increases the radioresistance of the microcolony-forming stem cells: protection is optimal with priming at 12 h (Phelps & Blackett, 1979).

The finding of either enhanced or reduced lung damage, according to the priming interval, might be explained in terms of the proliferating kinetics of the alveolar epithelium. Although difficult to relate to the slow cycling times of the unperturbed alveolar cells, they may be related to the kinetics of stimulated epithelial cells. Adamson & Bowden (1974) have studied the kinetics and morphology of epithelial regeneration following exposure to oxygen, which causes a proliferative cellular response, similar to that seen with cyclophosphamide, as well as with a number of other toxic agents. Type II epithelial cells showed mitotic activity and the peak mitotic activity occurred between the second and third day after stopping oxygen, as shown by mitotic arrest with colchicine and nuclear labelling with [$^3$H]TdR. The proliferating Type II cells were calculated to have a generation time of 3 days, and by 3–4 days after $O_2$ exposure lung morphology had returned to normal. On the basis of these results, our findings may be explained as follows. When the large dose of Cy follows the priming dose after 1 or 2 days, a large number of cells are in cycle and lung epithelium is particularly sensitive to the drug. If, however, the large dose is delayed to 7 days or more, cell division has declined and the resultant increased cell population renders the lung more tolerant to the large dose. It is difficult to relate the probable protection seen with priming at 12 h to cell kinetics, since for one thing Cy has not been reported to be phase-specific. However, protection could be due to non-kinetic factors, as have been proposed for cytosine arabinoside given 12 h before gut irradiation (Phelps & Blackett, 1979).

Another interesting effect of Cy on the lungs of mice, which also depends on the timing of its administration, is the enhanced lung-colony formation after i.v. injections of tumour cells (van Putten *et. al.*, 1975; Carmel & Brown, 1977; Steel & Adams, 1977). In the C3H mice, enhancement was optimal when the drug was given 24 h before the tumour cells (Carmel & Brown, 1977). CBA mice primed at the same interval in this study showed maximal enhancement of lung damage. It may therefore be that the condition of the lung which makes it more susceptible to Cy lung damage also makes it more susceptible to tumour implantation. Strain differences make such conclusions speculative at present, since in C57 mice the optimal time for Cy enhancement of lung colonies is 2–4 days (Steel & Adams, 1977). However, these particular mice may have delayed development of lung changes, since their median survivals following lung irradiation were between 200 and 310 days, whereas in CBA mice the medians ranged from 95 to 130 days, which are similar to the median survivals reported by other investigators (Steel *et al.*, 1979).

If the metabolism of Cy and the lung biology of man and mouse were similar, Cy lung damage in man would be reduced by priming with Cy 7 days before the large dose, and divided doses at intervals of between 1 and 7 days should perhaps be avoided. However, the cell kinetics of human lung and the metabolism of Cy are different in man and mouse; therefore the absolute time intervals may need modification. In addition man differs from mouse in that Cy-induced lung damage is unusual, presumably because dose-limiting toxicity develops in other organs first. None the less lung damage does occur, and its prevention would be of value.

We would like to thank Dr Nick Blackett, Dr Gordon Steel and Professor M. J. Peckham for helpful discussions and advice throughout this study. We thank Dr E. L. Travis for her assistance in reviewing the histological material, and Mrs Annabel Thomas for typing the script.

### REFERENCES

ADAMSON, I. Y. R. & BOWDEN, D. H. (1974) The type 2 cell as progenitor of alveolar epithelial regeneration. A cytodynamic study in mice after exposure to oxygen. *Lab. Invest.*, **30**, 35.

ANDRÉ, R., ROCHANT, H., DREYFUS, B., DUHAMEL, G. & PÉCHÈRE, J-CL. (1967) Fibrose interstitielle diffuse du paumon au cours d'une maladie de Hodgkin traitée par des doses élevées d'Endoxan. *Bull. Soc. Hôp. Med.* (Paris), **118**, 1133.

BERENBAUM, M. C. (1974) The production of pulmonary oedema in mice by cyclophosphamide and iodide. *Agents Actions*, **4**, 7.

BLACKETT, N. M. & AGUADO, M. (1979) The enhancement of haemopoietic stem cell recovery in irradiated mice by prior treatment with cyclophosphamide. *Cell Tissue Kinet.*, **12**, 291.

CARMEL, R. J. & BROWN, J. M. (1977) The effect of cyclophosphamide and other drugs on the incidence of pulmonary metastases in mice. *Cancer Res.*, **37**, 145.

GOULD, V. E. & MILLER, J. (1975) Sclerosing alveolitis induced by cyclophosphamide. Ultrastructural observations on alveolar injury and repair. *Am. J. Pathol.*, **81**, 513.

GREGORY, S. A., FRIED, W., KNOPSE, W. H. & TROBAUGH, F. E. (1971) Accelerated regeneration of transplanted haemopoietic stem cells in irradiated mice pretreated with cyclophosphamide. *Blood*, **37**, 196.

JENEY, A., CONNORS, T. A. & JONES, M. (1968) The toxicity of merophan after pretreatment with subtoxic doses. *Acta Physiol. Acad. Sci. Hung.*, **33**, 89.

MARK, G. J., LEHIMGAR-ZADEH, A. & RAGSDALE, B. D. (1978) Cyclophosphamide pneumonitis. *Thorax*, **33**, 89.

MILLAR, J. A., HUDSPITH, B. N. & BLACKETT, N. M. (1975) Reduced lethality in mice receiving a combined dose of cyclophosphamide and busulphan. *Br. J. Cancer*, **32**, 193.

MILLAR, J. L. & HUDSPITH, B. N. (1976) Sparing effect of cyclophosphamide (N.S.C.-26271) pretreatment on animals lethally treated with $\gamma$-irradiation. *Cancer Treat. Rep.*, **60**, 409.

MILLAR, J. L. & MCELWAIN, T. J. (1978) Combinations of cytotoxic agents that have less than expected toxicity on normal tissues of mice. In *Antibiotics and Chemotherapy*. Ed. Schönfeld *et al.* Basel: Karger. p. 271.

O'CONNELL, T. X. & BERENBAUM, M. C. (1974) Cardiac and pulmonary effects of high doses of cyclophosphamide and isophosphamide. *Cancer Res.*, **34**, 1586.

PATEL, A. R., SHAH, P. C., RHEE, H. L., SASSOON, H. & RAO, K. P. (1976) Cyclophosphamide therapy and interstitial pulmonary fibrosis. *Cancer*, **38**, 1542.

PHELPS, T. A. & BLACKETT, N. M. (1979) Protection of intestinal damage by pretreatment with cytarabine (cytosine arabinoside). *Int. J. Radiat. Oncol. Biol. Phys.*, **5**, 1617.

PHILLIPS, T. L. & FU, K. K. (1976) Quantification of combined radiation therapy and chemotherapy effects on critical normal tissues. *Cancer*, **37**, 1186.

SOSTMAN, H. D., MATTHAY, R. A. & PUTMAN, C. E. (1977) Cytotoxic drug-induced lung disease. *Am. J. Med.*, **62**, 608.

STEEL, G. G. & ADAMS, K. (1977) Enhancement by cytotoxic agents of artificial pulmonary metastases. *Br. J. Cancer*, **36**, 653.

STEEL, G. G., ADAMS, K. & PECKHAM, M. J. (1979) Lung damage in C57BL mice following thoracic irradiation: enhancement by chemotherapy. *Br. J. Radiol.*, **52**, 741.

TRAVIS, E. L., VOJNOVIC, B., DAVIES, E. E. & HIRST, D. G. (1979*a*) A plethysmographic method for measuring function in locally irradiated mouse lung. *Br. J. Radiol.*, **52**, 67.

TRAVIS, E. L., DOWN, J. D. & HOLMES, S. J. (1979*b*) Breathing frequency as a measure of acute and late radiation damage in mouse lungs. *Int. J. Radiat. Oncol. Biol. Phys.*, **5** (Supp. 2), 85.

VAN PUTTEN, L. M., KRAM, L. K. J., VAN DIERENDONCK, H. H. C., SMINK, T. & FÜZY, M. (1975) Enhancement by drugs of metastatic lung nodule formation after intravenous tumour cell injection. *Int. J. Cancer*, **15**, 588.


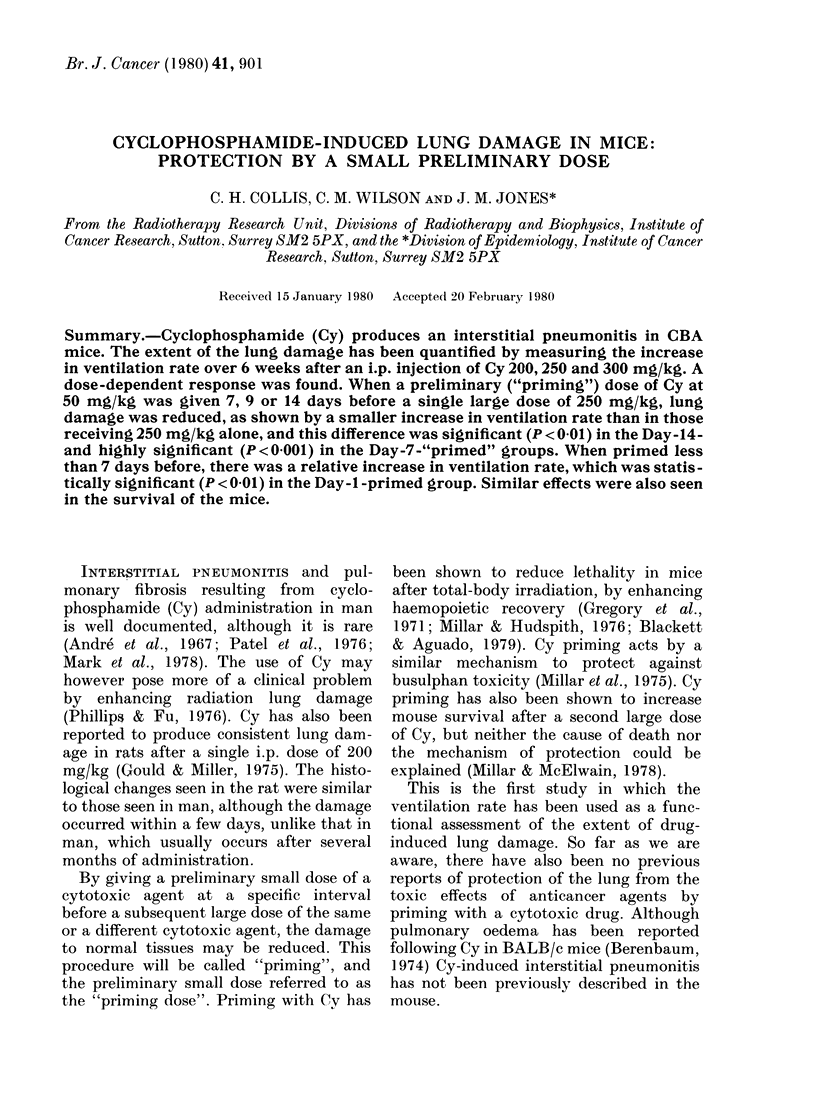

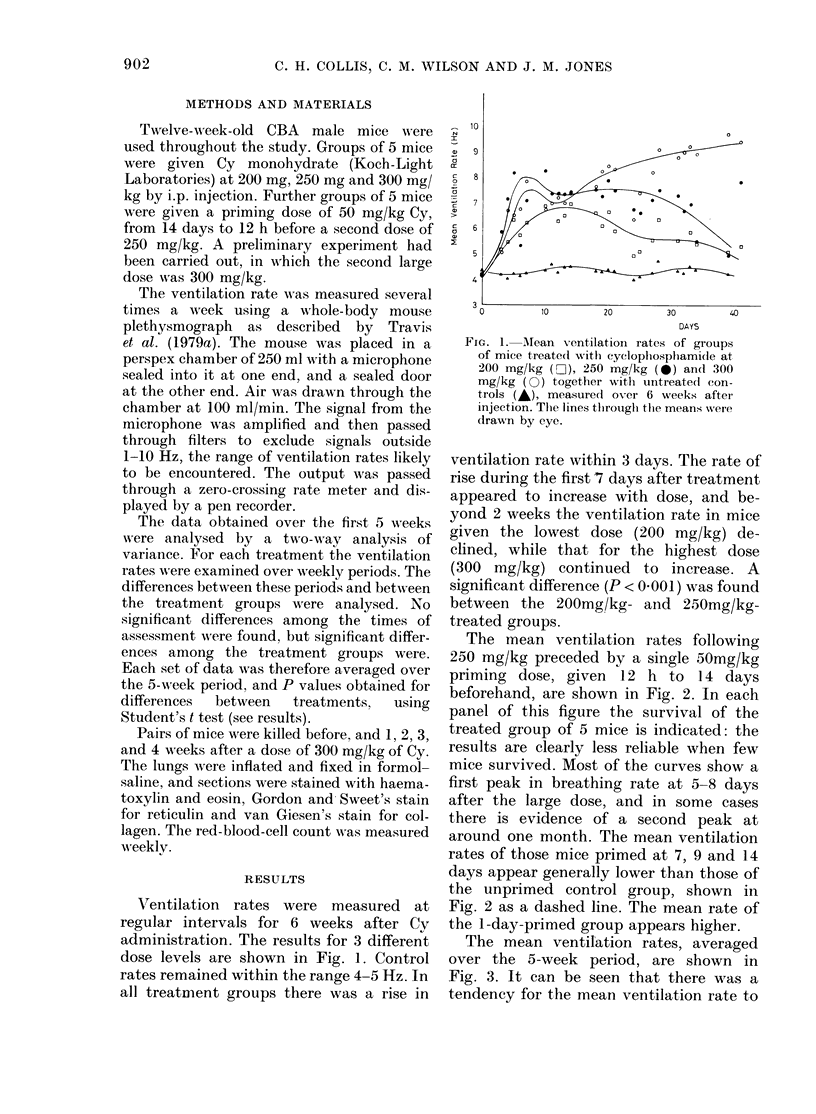

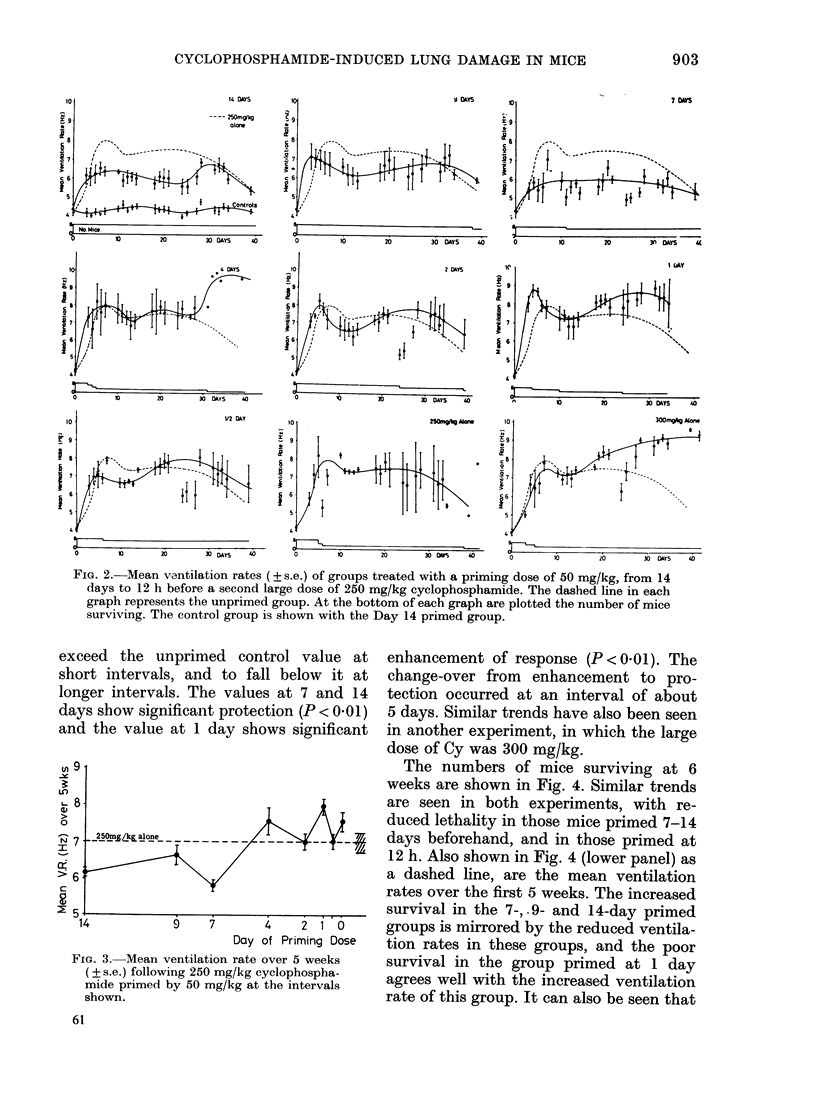

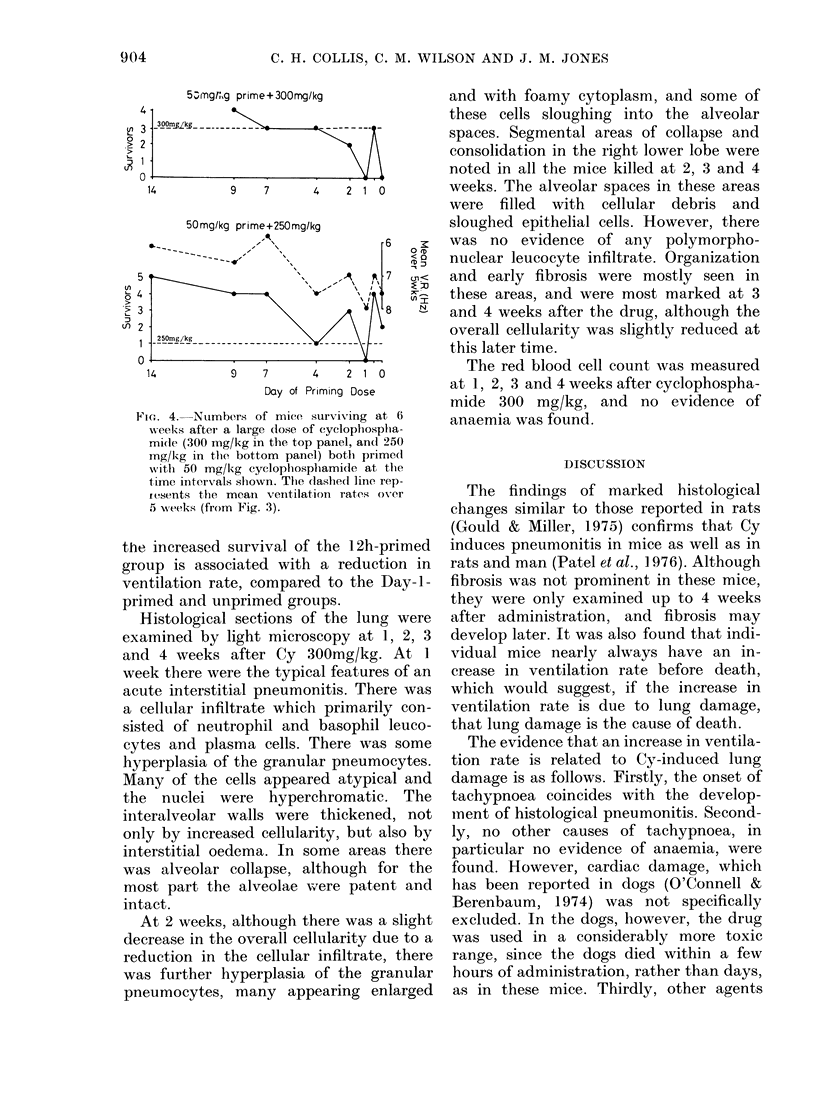

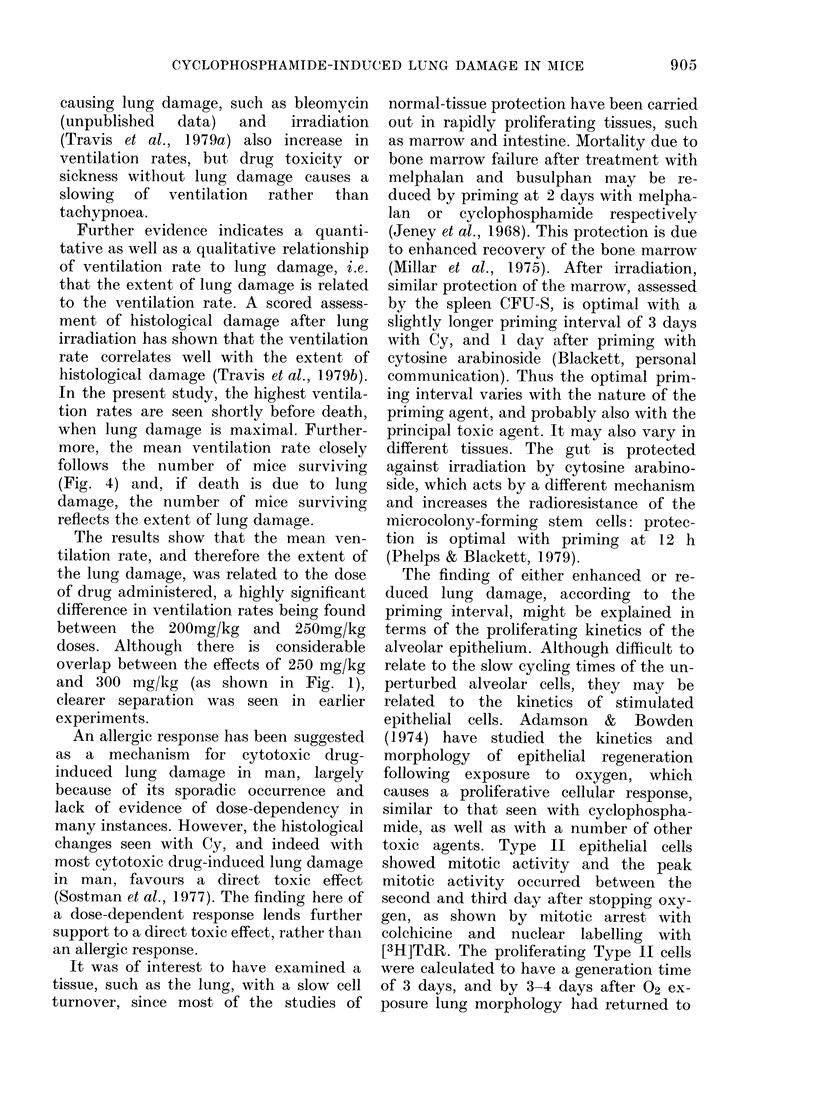

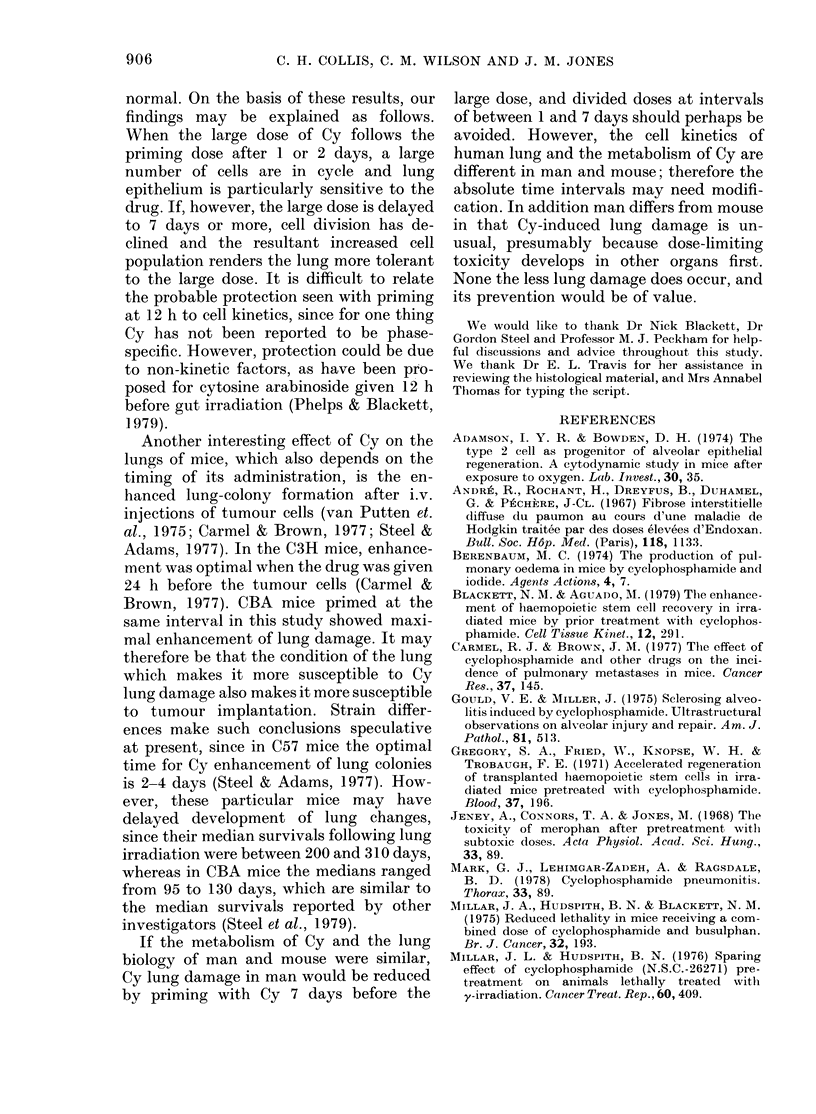

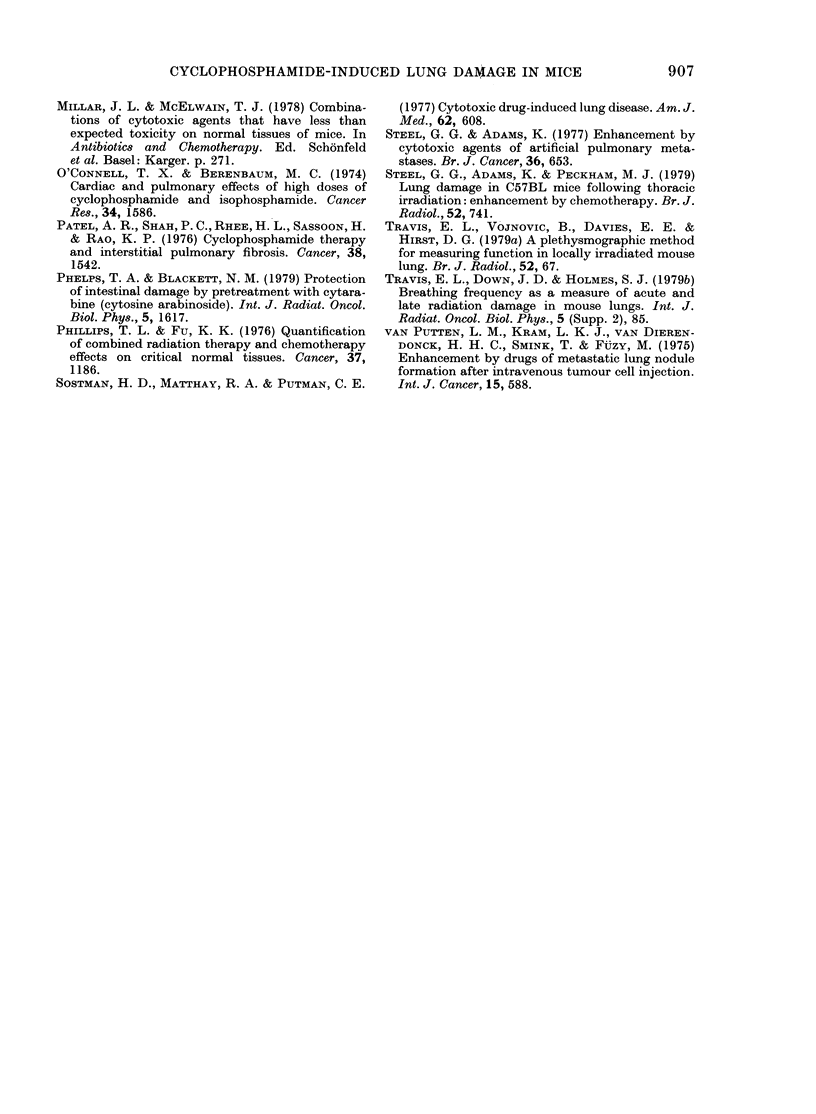


## References

[OCR_00639] Adamson I. Y., Bowden D. H. (1974). The type 2 cell as progenitor of alveolar epithelial regeneration. A cytodynamic study in mice after exposure to oxygen.. Lab Invest.

[OCR_00645] André R., Rochant H., Dreyfus B., Duhamel G., Péchère J. C. (1967). Fibrose interstitielle diffuse du poumon au cours d'une maladie de Hodgkin traitée par des doses élevées d'endoxan.. Bull Mem Soc Med Hop Paris.

[OCR_00657] Blackett N. M., Aguado M. (1979). The enhancement of haemopoietic stem cell recovery in irradiated mice by prior treatment with cyclophosphamide.. Cell Tissue Kinet.

[OCR_00663] Carmel R. J., Brown J. M. (1977). The effect of cyclophosphamide and other drugs on the incidence of pulmonary metastases in mice.. Cancer Res.

[OCR_00669] Gould V. E., Miller J. (1975). Sclerosing alveolitis induced by cyclophosphamide. Ultrastructural observations on alveolar injury and repair.. Am J Pathol.

[OCR_00677] Gregory S. A., Fried W., Knospe W. H., Trobaugh F. E. (1971). Accelerated regeneration of transplanted hematopoietic stem cells in irradiated mice pretreated with cyclophosphamide.. Blood.

[OCR_00688] Mark G. J., Lehimgar-Zadeh A., Ragsdale B. D. (1978). Cyclophosphamide pneumonitis.. Thorax.

[OCR_00693] Millar J. L., Hudspith B. N., Blackett N. M. (1975). Reduced lethality in mice receiving a combined dose of cyclophosphamide and busulphan.. Br J Cancer.

[OCR_00699] Millar J. L., Hudspith B. N. (1976). Sparing effect of cyclophosphamide (NSC-26271) pretreatment on animals lethally treated with gamma-irradiation.. Cancer Treat Rep.

[OCR_00714] O'Connell T. X., Berenbaum M. C. (1974). Cardiac and pulmonary effects of high doses of cyclophosphamide and isophosphamide.. Cancer Res.

[OCR_00720] Patel A. R., Shah P. C., Rhee H. L., Sassoon H., Rao K. P. (1976). Cyclophosphamide therapy and interstitial pulmonary fibrosis.. Cancer.

[OCR_00726] Phelps T. A., Blackett N. M. (1979). Protection of intestinal damage by pretreatment with cytarabine (cytosine arabinoside).. Int J Radiat Oncol Biol Phys.

[OCR_00732] Phillips T. L., Fu K. K. (1976). Quantification of combined radiation therapy and chemotherapy effects on critical normal tissues.. Cancer.

[OCR_00738] Sostman H. D., Matthay R. A., Putman C. E. (1977). Cytotoxic drug-induced lung disease.. Am J Med.

[OCR_00743] Steel G. G., Adams K. (1977). Enhancement by cytotoxic agents of artificial pulmonary metastasis.. Br J Cancer.

[OCR_00748] Steel G. G., Adams K., Peckham M. J. (1979). Lung damage in C57B1 mice following thoracic irradiation: enhancement by chemotherapy.. Br J Radiol.

[OCR_00760] Steel G. G., Peckham M. J. (1979). Exploitable mechanisms in combined radiotherapy-chemotherapy: the concept of additivity.. Int J Radiat Oncol Biol Phys.

[OCR_00754] Travis E. L., Vojnovic B., Davies E. E., Hirst D. G. (1979). A plethysmographic method for measuring function in locally irradiated mouse lung.. Br J Radiol.

[OCR_00768] van Putten L. M., Kram L. K., van Dierendonck H. H., Smink T., Füzy M. (1975). Enhancement by drugs of metastatic lung nodule formation after intravenous tumour cell injection.. Int J Cancer.

